# 

**Published:** 1856-01

**Authors:** 


### CONTENTS OF NO. I, VOL. III.

### ORIGINAL COMMUNICATIONS.

Page.

Introductory Lecture to the Class of the Iowa University.—By Prof. J. R. Allen,.... 1

Outraged Laws of Hygiene.—By Prof. D. L. M'Gugin.... 14

Case of Compound Comminuted Fracture. Keokuk City Hospital Report—By A. M. Carpenter... 20

A Case of Paraplegia—By P. Van Patten.... 30

Cases in Practice—By J. C. Hinsey. 32

Case in Private Practice—By Jno. R. Allen.... 42

### DEPARTMENT OF SELECTIONS.

Lecture on Fever—By Wm Stokes. 44

The Physiology of the different varieties of Paralysis—By Marshall Hall.... 52

On the use of Tinc. Ferr. Chlorid. in Uterine Hemorrhages—By Frederick Schreier.... 54

Rules for the Administration of Chloroform in Labor—By Murphy. 56

### EDITORIAL DEPARTMENT.

Empirical Publications.... 58

The Pardon of Dr. Beale.... 63

Socialistic Theories, in their Medical Aspect.... 65

Rheumatism—By E. S. S. Rouse.. 66

On Topical Medication of the Larynx.... 67

Present College Session.... 68

Our Journal.... 70

Agricultural Chemistry.... 71

Election.... 72

Surgical Operations.... 72

Page

Dental Surgery.... 73

Proceedings of the State Society... 73

### BIBLIOGRAPHICAL NOTICES.

New Journal.... 74

The modern treatment of Syphilitic Diseases, both Primary and Secondary—By Langster Parker.. 74

The principles and practice of Obstetrical Medicine and Surgery. By Francis H. Ramsbatham.... 75

The Book of Prescriptions—By Henry Beasley.... 75

An enquiry into the Pathological Importance of Ulceration of the Os Uteri—By Charles West.... 75

Materia Medica or Pharmacology and Therapeutics—By William Tully.... 76

Elements of Medicine, &c—By S. H. Dicksen.... 76

Introductory of Prof. Brainard.... 77

Clinical Lectures on Surgery—By M. Nelaton.... 78

Transactions of the American Medical Association.... 79

Principles of Human Physiology—By William B. Carpenter.... 79

Foster's Chemistry.... 79

Constitution and By-Laws of the American Medical Association.. 80

The Causes and Prevention of Yellow Fever—By E. H. Barton.... 80

A Treatise on Epidemic Cholera—By Horatio Gates Jameson, Sr.. 80

Transactions of the New Hampshire Medical Society.... 80

Hymeneal.... 80

